# Supplementary material for: Antifungal Activity of 2-Allylphenol Derivatives on the Botrytis cinerea Strain: Assessment of Possible Action Mechanism
Source: Int J Mol Sci. 2023 Mar 31;24(7):6530. doi: 10.3390/ijms24076530 (PMC10095406; doi:10.3390/ijms24076530)
Supplement: Supplementary file 1 [file ijms-24-06530-s001.zip › ijms-2293002-supplementary.pdf]

Article

Antifungal Activity of 2-Allylphenol Derivatives on the *Botrytis cinerea* Strain: Assessment of Possible Action Mechanism

Andres F. Olea [1], Julia Rubio [1], Claudia Sedan [1], Denisse Carvajal [1], Maria Nuñez [2], Luis Espinoza [2], Ligia Llovera [2], Gerard Nuñez [2], Lautaro Taborga [2,*] and Héctor Carrasco [1,*]

[1] Grupo QBAB, Instituto de Ciencias Químicas Aplicadas, Facultad de Ingeniería, Universidad Autónoma de Chile, San Miguel, Santiago 8900000, Chile

[2] Departamento de Química, Universidad Técnica Federico Santa María, Avenida España 1680, Valparaíso CP 2340000, Chile

* Correspondence: lautaro.taborga@usm.cl (L.T.); hector.carrasco@uautonoma.cl (H.C.)

Supplementary Materials: The following supporting information can be downloaded at: www.mdpi.com/xxx/s1, **Figure S1**: 1D-NMR spectra of 2-allyl-1-methoxy-4-nitrobenzene (**11**); **Figure S2:** 1D-NMR spectra of 2-allyl-1-methoxy-4-nitrobenzene (**11**).**Figure S3**: MS (EI+) Chromatogram and spectrum of 2-allyl-1-methoxy-4-nitrobenzene (**11**); **Table S1**: Primers (completing description)

^{1}H-NMR

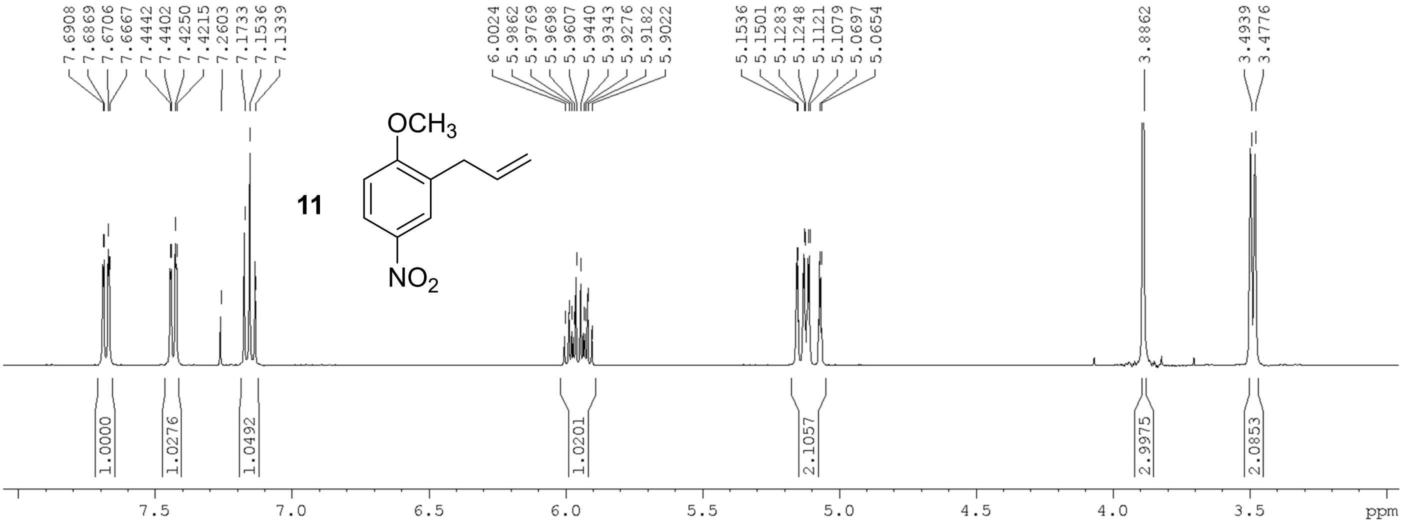

^{13}C-NMR

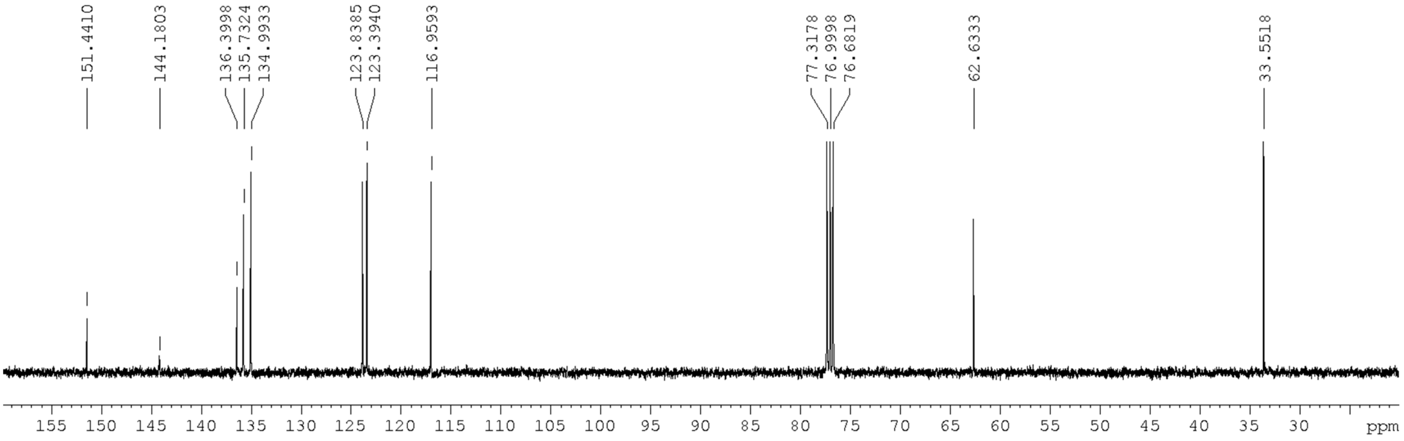

Dept-135

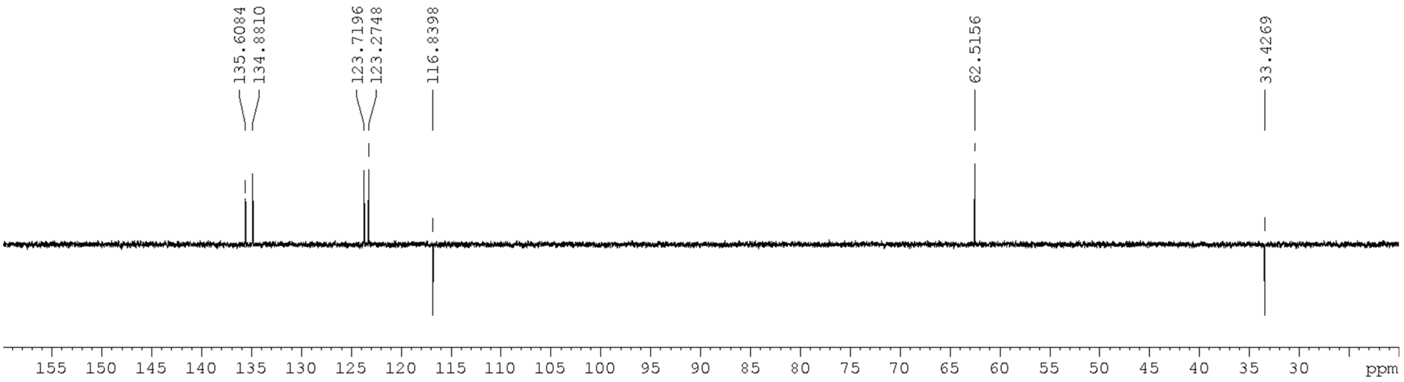

Figure S1: 1D-NMR spectra of 2-allyl-1-methoxy-4-nitrobenzene (**11**).

2D-HSQC

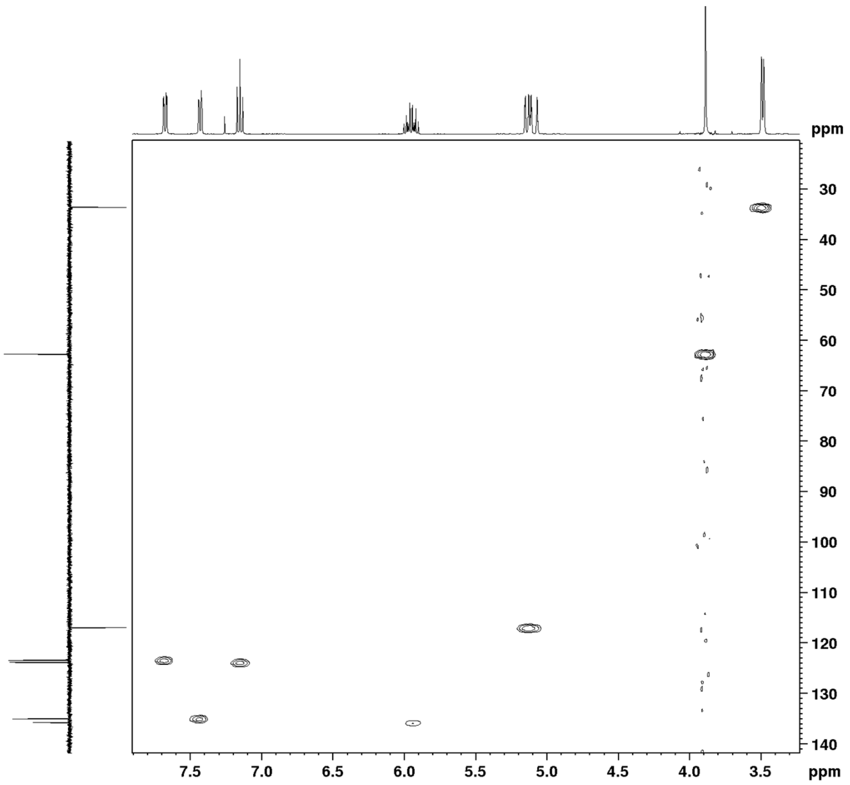

11

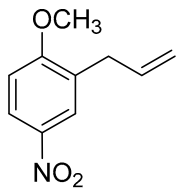

2D-HMBC

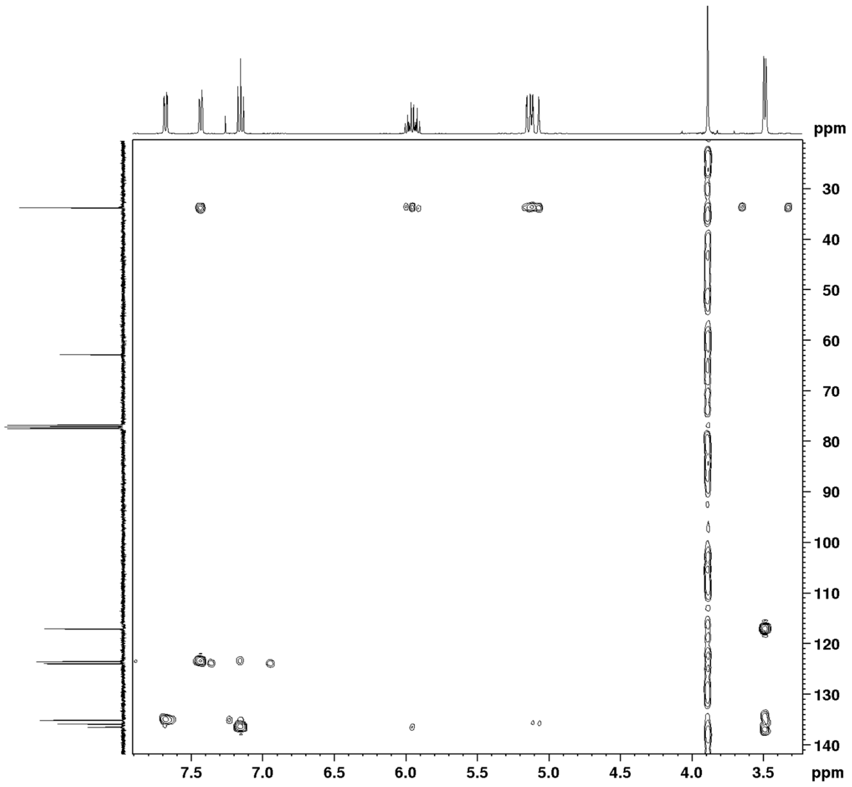

11

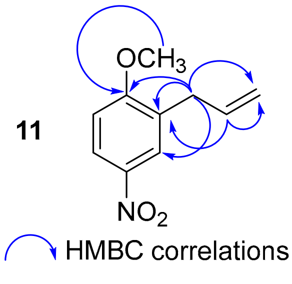

Figure S2: 2D-NMR spectra of 2-allyl-1-methoxy-4-nitrobenzene (**11**).

Chromatogram: retention time 9.665 min.

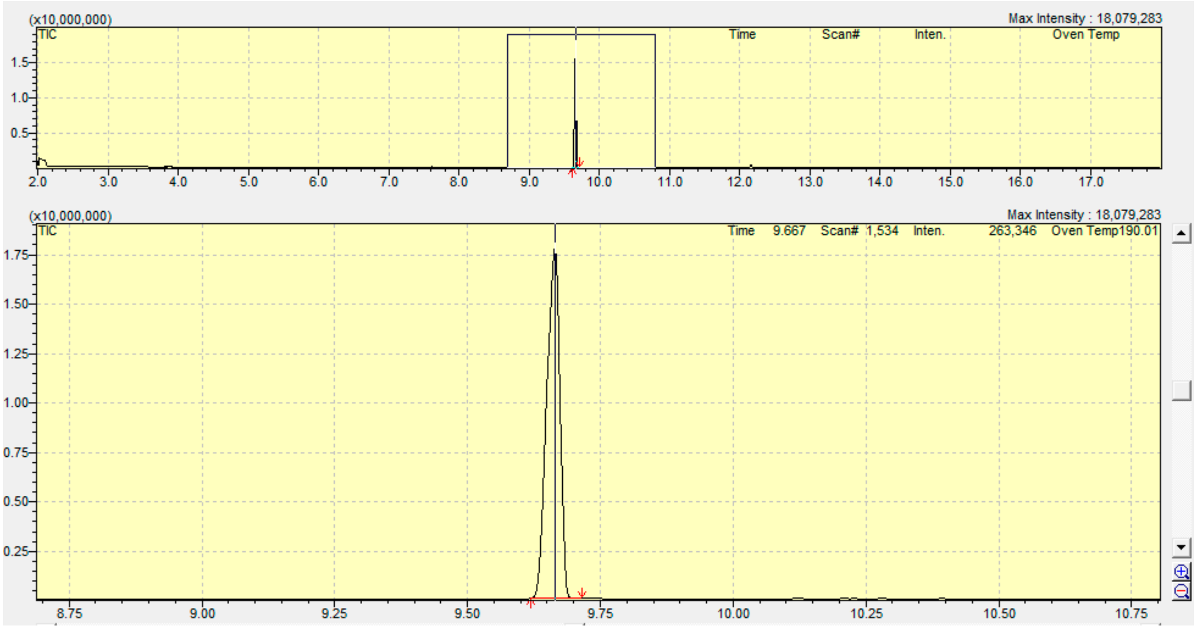

[M+] 193 m/z

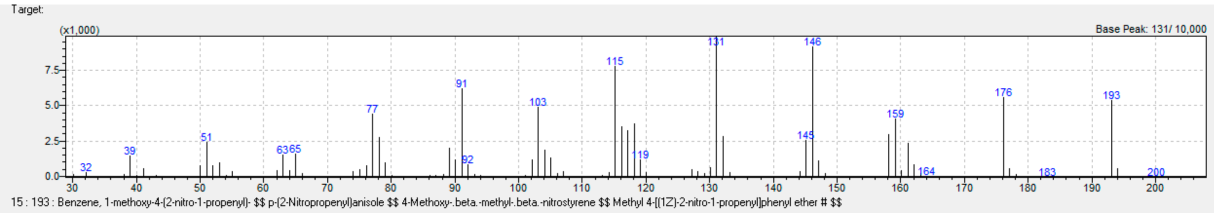

Figure S3: GC/MS (EI$^+$) Chromatogram and spectrum of 2-allyl-1-methoxy-4-nitrobenzene (**11**).

Table S1: Primers

Primers	Sequences	Tm °C	Primer's efficiency
cas-1 F	5'-ATGGAGGCTATGGAGCACCA-3'	57	97
cas-1 R	5'-GCAACACCGCTCATATCACCT -3'		
Bcaox F	5'-CAATGTATGTATCAAGGGTA-3'	57	99
Bcaox R	5'-TCTTCATGCGATCCCTCA-3'		
Bchex F	5'-TCTACTTCAACGAGGCTTC-3'	57	99
Bchex R	5'-CACCAGATTGACCGAAAAC-3'		
BcUBcE F	5'- CATCAACTCCAACGGAAGCA-3'	57	98
BcUbcE R	5'- TCGGTCGGTCTTGTAAACGT-3'		
